# Leveraging Mobile-Based Sensors for Clinical Research to Obtain Activity and Health Measures for Disease Monitoring, Prevention, and Treatment

**DOI:** 10.3389/fdgth.2022.893070

**Published:** 2022-06-14

**Authors:** Hari G. Dandapani, Natalie M. Davoodi, Lucie C. Joerg, Melinda M. Li, Daniel H. Strauss, Kelly Fan, Talie Massachi, Elizabeth M. Goldberg

**Affiliations:** ^1^Brown University, Providence, RI, United States; ^2^Department of Emergency Medicine, The Warren Alpert Medical School of Brown University, Providence, RI, United States

**Keywords:** smartphones, Researchkit, Apple Watch, falls, mobile applications, Research Electronic Data Capture (REDCap)

## Abstract

Clinical researchers are using mobile-based sensors to obtain detailed and objective measures of the activity and health of research participants, but many investigators lack expertise in integrating wearables and sensor technologies effectively into their studies. Here, we describe the steps taken to design a study using sensors for disease monitoring in older adults and explore the benefits and drawbacks of our approach. In this study, the Geriatric Acute and Post-acute Fall Prevention Intervention (GAPcare), we created an iOS app to collect data from the Apple Watch's gyroscope, accelerometer, and other sensors; results of cognitive and fitness tests; and participant-entered survey data. We created the study app using ResearchKit, an open-source framework developed by Apple for medical research that includes neuropsychological tests (e.g., of executive function and memory), gait speed, balance, and other health assessments. Data is transmitted via an Application Programming Interface (API) from the app to REDCap for researchers to monitor and analyze in real-time. Employing the lessons learned from GAPcare could help researchers create study-tailored research apps and access timely information about their research participants from wearables and smartphone devices for disease prevention, monitoring, and treatment.

## Introduction

Eighty-five percent of adults in the United States (US) own a smartphone, a marked increase from just 35% a decade ago (1). Researchers can harness smartphones as a rich source of data for clinical research (2), but a system to develop and deploy applications (apps), and a plan for data management and analysis, is essential to leveraging this data. One promising solution is Apple's ResearchKit, an open-source app-development framework for medical research (3), which allows investigators to create research apps to enroll participants, conduct remote surveys, and collect sensor-based quantitative data on participants' cognitive and motor performance.

ResearchKit-based mobile apps can collect both participant-entered data and information from sensors on the iPhone or wearable smart devices, such as the Apple Watch. (3). Researchers have used ResearchKit to conduct nevus measurements for melanoma (4), sexually transmitted infection risk questionnaires (5), and surveys on rheumatoid arthritis symptoms (6). Harnessing data from accelerometers, gyroscopes, and other physiological sensors via ResearchKit requires a multidisciplinary team with skills in data management, software engineering, and clinical research. Thus, uptake has been slow, and there is a lack of research detailing how to initiate this work and use ResearchKit-based mobile apps to conduct clinical research.

Additional barriers to widespread use may also include investigator concerns surrounding selection bias—individuals who use wearables and smartphones are generally younger, White, and affluent—as well as concerns surrounding data sharing and security (1, 7). These potential concerns can be addressed at the design stage of the study, allowing the research team to proceed with the research in a way that respects the privacy and anonymity of research participants and is in compliance with regulatory agencies.

Our objective is to provide a detailed overview of how to approach the design of a study using wearable sensors. Specifically, we enumerate how a ResearchKit study app can be used to leverage sensor data from the Apple Watch and participant-entered survey data on the iPhone for clinical research (8, 9). We share our experiences using ResearchKit for a study on geriatric fall prevention: the Geriatric Acute and Post-acute Fall Prevention Intervention II (GAPcare II) study (8). The lessons we learned from GAPcare II will be helpful to other researchers as they consider the use of wearables and smartphone devices to collect real-time data about research participants through study-tailored research apps.

### Materials and Methods

#### Summary

GAPcare II is a research study aimed at reducing subsequent falls among older adults ages 65 and older who present to the emergency department (ED). In this study, we used the Apple Watch to collect cognitive and fitness measures, and to track fall occurrences. After creating our app, our first step was to field test our research app among a small number of older adults to obtain feedback on our app design and refine our study procedures. Subsequently, we used the iPhone and Apple Watch to collect outcome data from a larger group of participants enrolled in a clinical trial aiming to reduce recurrent falls. Here we detail the steps we followed and lessons learned from the field testing.

**Figure 1:**
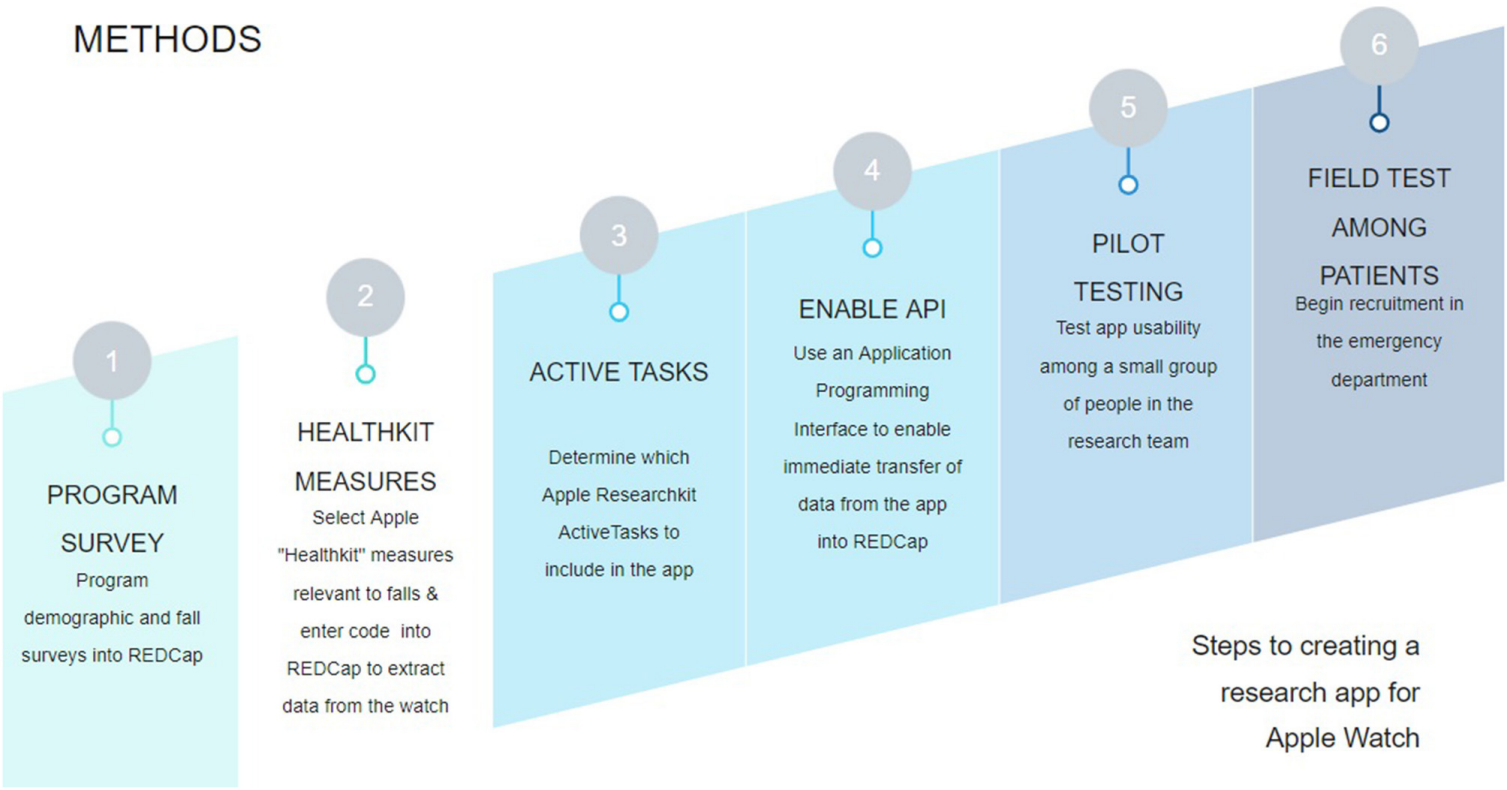
High-level study workflow for the GAPcare II study. We began by creating the surveys for the relevant fields in REDCap. Then, we programmed the app to send the relevant measurements from HealthKit, which measures health data in the iPhone and Apple Watch. We then determined which Active Tasks to include in our study based on our study goals. Then we enabled the REDCap API, allowing members of the research team to perform tests to verify that data collected from the app was sent to REDCap. Once we verified that the app worked as intended, we began field testing the app with older adult patients from the ED.

To field test the research app, we recruited participants who recently experienced a fall from the ED. After participants provided their consent, research assistants (RAs) followed a standardized protocol to orient them to the devices used in the study and our research app: the RI FitTest App. Following this orientation, participants put on a study-provided Apple Watch and performed a series of cognitive and motor Active Tasks guided both by our RAs and instructions from the app. While the participant completed the Active Tasks, the app passively collected several physiological measurements from the iPhone and Apple Watch sensors, including heart rate and step count. Participants were asked to wear the Apple Watch and use the research app for 30 days, or until they were no longer able to participate. After completing the field test, RAs completed semi-structured, qualitative interviews with participants to elicit their perspectives on the usability of the technology used in the study, as well as suggestions for improvement for the research app. Data collected by the app was transmitted to REDCap (Research Electronic Data Capture)—an online HIPAA-compliant database for medical research—via an Application Programming Interface (API). Figure 1 summarizes our workflow for the GAPcare II study, from the initial programming and set up of the RI FitTest App to field testing the app with patients from the ED.

#### ResearchKit

Apple ResearchKit is a tool to help investigators develop apps to track health indicators, survey participants, and collect physiological data from HealthKit sensors in the Apple Watch and iPhone. ResearchKit also contains pre-programmed modules for electronic informed consent and modules with cognitive and motor assessments, called “Active Tasks.”

Active Tasks use device sensors such as the accelerometer, gyroscope, and microphone to collect data. Participants may be prompted to walk, speak into a microphone, or perform memory tests depending on the task. Researchers can choose which Active Tasks to include for their specific study. We chose Active Tasks for GAPcare II that could provide insights into the reasons why an individual may fall. The Principal Investigator of the study (EG) met with a neuropsychologist with experience in geriatric assessments to decide which Active Tasks would have the greatest relevance to falls. For instance, the Trail Making test (described below) was chosen because it provides insights into deficits in visual attention and task switching, which could lead to falls. The five Active Tasks included in our app are described below (9).

##### Gait and Balance

The participant walks in a straight line for 20 steps then turns around and walks back 20 steps. Then, the participant stands still for 5 s. The gait and balance Active Task allows the researcher to measure stride length, smoothness, sway, and other characteristics of the participant's walk.

##### Timed Walk

The participant walks in a straight line as quickly as possible and then turns around to walk back in the opposite direction for the same distance. The GPS and gyroscope within the smartphone allow the participant's location, pedometer data, and device movement to be tracked to obtain a gait measurement.

##### Reaction Time

The participant is asked to shake the iPhone immediately after noticing a large dot that appears on the screen. This task is divided into rounds of varying lengths of time between dot appearance, which allows the researcher to evaluate reaction time to visual stimuli. The Active Task continues until the participant completes five rounds.

##### Trail Making Test

The participant starts by tapping 1, then A, and subsequently 2, then B, alternating between sequential numbers and letters until they reach 7. The trail making test evaluates visual attention and task-switching ability by recording the time required to tap a series of dots in ascending order, alternating between letters and numbers.

##### Stroop Test

Participants look at color words, such as blue, red, or green, and are asked to ignore the actual meaning of the word, and instead tap on the color that it is shown in (e.g., the participant may see the word “green” in blue font and must select blue rather than green). In other words, the participant is required to perform a less automated task (e.g., naming the font color) while inhibiting the interference arising from a more automated task (e.g., reading the word). The Stroop Test examines multiple cognitive functions: the ability to inhibit cognitive interference, processing speed, cognitive flexibility, and working memory.

#### Apple Watch Sensors

In GAPcare II, we employ the Apple Watch Series 4, which received clearance from the Food and Drug Administration as a Class II medical device in 2018 for its ability to record an ECG (10). We chose to use the Apple Watch because it passively records fall occurrences, making it useful in a population that may forget they have fallen, and because studies have demonstrated that its sensors are accurate in recording step counts and heart rate data, which could provide valuable insights on why falls are occurring in our participants. Previous studies have found that the Apple Watch had the best performance among seven wrist-worn monitoring devices reporting heart rate under controlled laboratory conditions of walking, running, and cycling (11). Additionally, a validation study evaluating step count obtained from the Apple Watch found that the device recorded daily step counts for adults across different BMI categories and age groups with high accuracy (12).

Because the Apple Watch enables the collection of longitudinal data on falls, physiological inputs, and motor functioning from participants, it could provide insight into the precipitating events before a fall occurs, as well as the cognitive and motor consequences after a fall event. To date, these factors remain poorly understood and have hampered the development of fall prevention programs tailored to addressing the causes of falls (13).

#### REDCap

REDCap is a secure, HIPAA-compliant online web application used to store and manage surveys and databases that is available at no cost to most researchers at academic medical centers (14). REDCap allows teams of researchers to create forms storing quantitative data on the health information of study participants. Its availability as a web application allows different members of the research team to access the service with varying levels of permissions across multiple projects. Additionally, data collection instruments can be shared among research teams from different universities.

While it is possible to enter data into REDCap manually, researchers can also use the REDCap API, which allows other web-connected technologies to input and retrieve data from the online database. To enable a researcher to use the REDCap API for their project, the owner of the database must generate an API key—a secure string of random letters and digits—and share it with the other service that provides data for input or retrieves data from REDCap. For instance, the researcher could write a program that uses the API key to send secure requests to the REDCap server (15, 16). The API allows for basic data import and export that can be performed programmatically, as well as other more complex functionality, such as project creation and user rights management (17).

RI FitTest uses status/post—a login and authentication tool built with Apple's secure CloudKit software by researcher Dr. Christopher Metts—to authenticate users of the app and manage login sessions via the API (18). Status/post integrates an academic institution's REDCap project with ResearchKit-generated apps, allowing iPhone and Apple Watch data to be matched with each study participant. This platform enables the research team to create unique app login codes and REDCap IDs for each participant, ensuring that the app is only able to access data relevant to its associated participant. Each participant retains their login and REDCap ID for the entire study period, which enables the research team to longitudinally track and analyze their data.

When RI FitTest detects information that needs to be sent to REDCap, it can use the information about the participant—managed by status/post—and the API to send a request directly to REDCap. When the data arrives in REDCap, it appears as an entry in the database, which members of the research team can then access. Researchers can then easily access the data or download it for analysis from REDCap. REDCap allows data to be downloaded as a CSV, STATA, R, or SAS file.

## Results

### Pilot Testing Among Participants

After creating our research app and deciding on the initial workflow for our project, we recruited participants for the field test. During the field test, we oriented participants to the technology, had them perform surveys and Active Tasks for 30 days or until they were no longer able to perform them, and subsequently interviewed participants on their experiences. We gained valuable information about the app's design and our procedures from participants, which allowed us to modify our app and workflow to better accommodate participants and improve the quality of data collected. We share the workflow and lessons learned in the following sections.

### Recruitment of Study Participants and Technology Orientation

RAs screened patients in the ED, reviewing electronic health records to ensure they met inclusion criteria. Then, RAs approached potential participants and, if they were interested, obtained consent for enrollment in GAPcare II. For every prospective participant screened, an RA used the REDCap everyday carry software on an iPad to enter screening information and demographics.

After obtaining consent, the RA created an account in status/post and a subject ID in REDCap, which the research team used to access and analyze data from that participant. The research team then collected information from participants about past falls, health history, and current cognitive and functional status. This data was manually entered by research staff into surveys in REDCap. After collecting this data, the RA oriented the participant to the iPhone and Apple Watch by following a training guide (8) and demonstrating basic functionality.

Subsequently, participants were guided through the required Active Tasks. Data from this encounter was stored in two separate projects within REDCap: one for manually entered data about the participant (e.g., surveys about their health history and past falls), and a second for data from the Apple Watch and iPhone via the REDCap API. The research team maintained a separate document to relate both data sources to the participant using the participant's app login and the participant's REDCap subject ID.

### Active Tasks

When designing the app, the researcher can specify how frequently Active Tasks should be performed and set up push notifications to remind the participant to complete them. Specifically, participants were asked to perform Active Tasks during the initial ED visit and subsequent follow-up visits.

**Figure 2:**
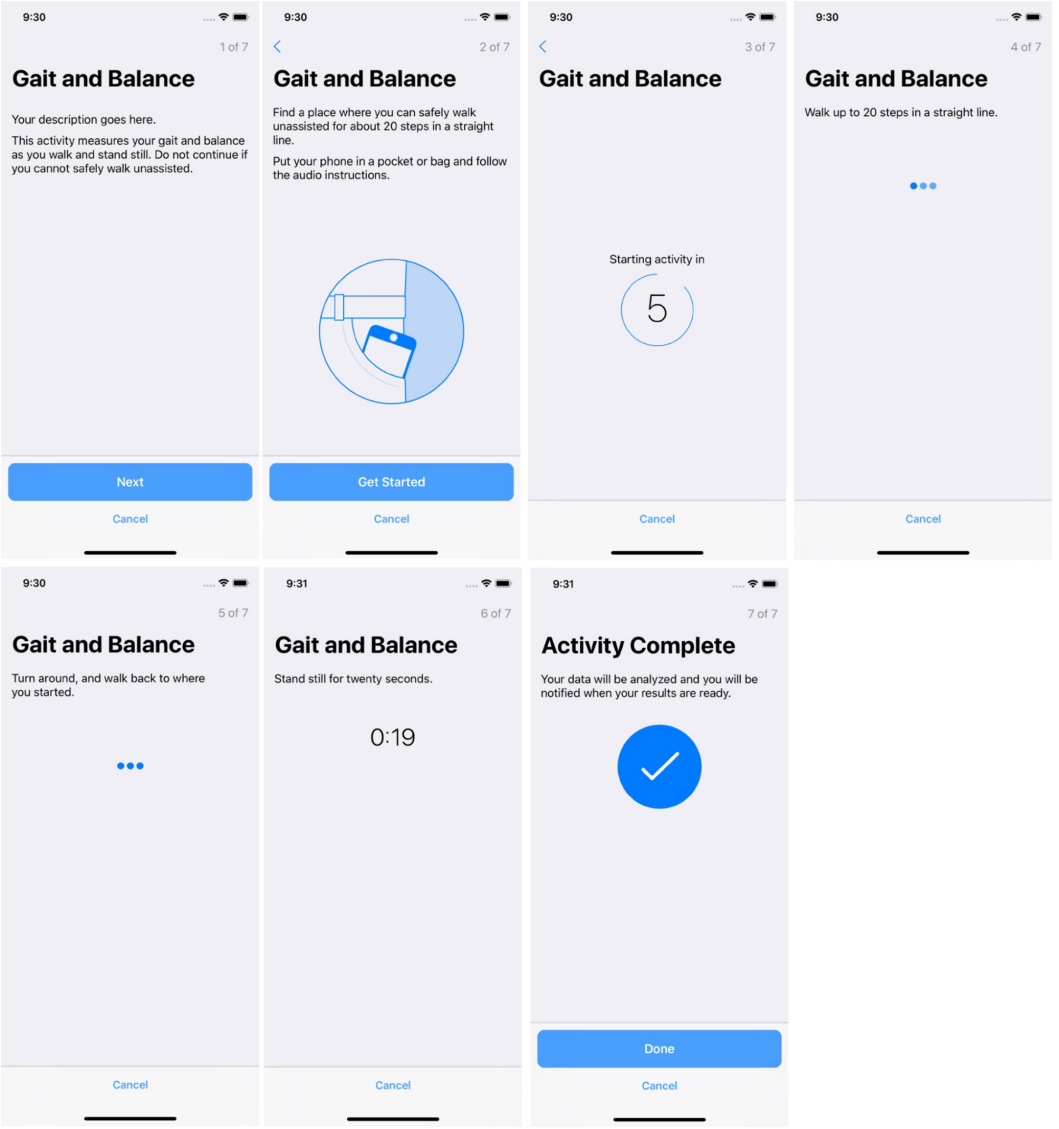
Screenshots from the ResearchKit active task: Gait and balance.

**Figure 3:**
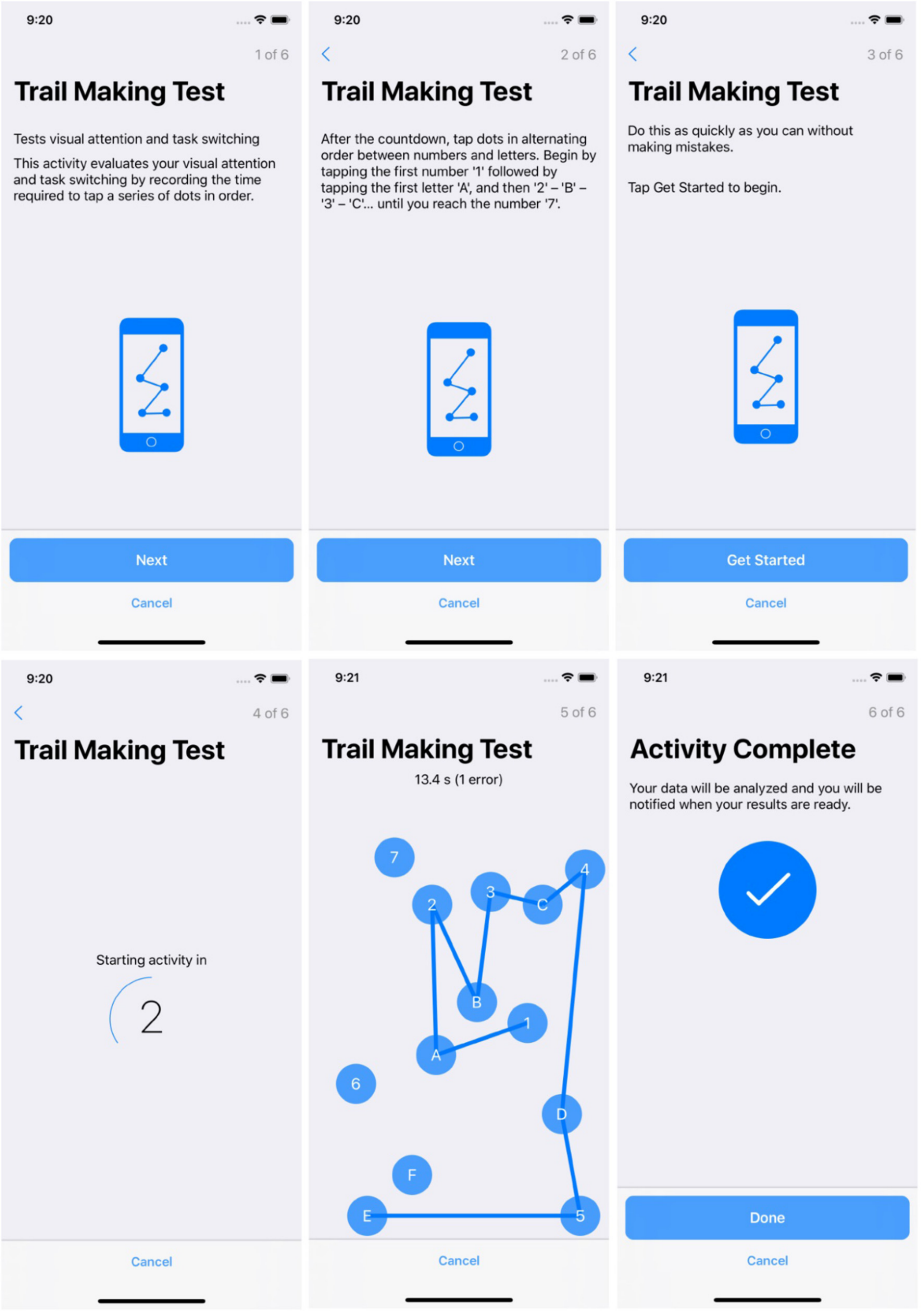
Screenshots from the ResearchKit active task: trail making test.

Figures 2, 3 show participant-facing instructions for the gait and balance task and trail making test, respectively.

Screenshots from the gait and balance Active Task are included in Figure 2.

Screenshots from the trail making Active Task are included in Figure 3.

### Using and Accessing REDCap

Members of the research team used REDCap throughout the study to monitor participants' progress and perform quality checks.

**Figure 4:**
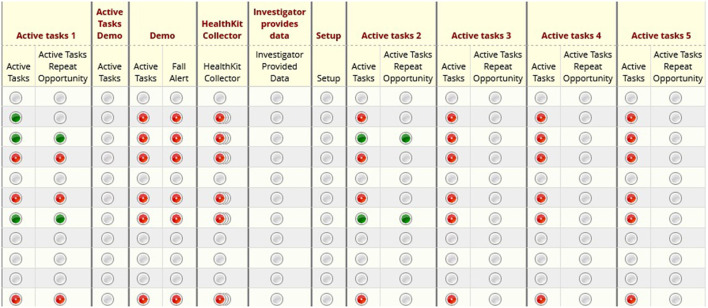
REDCap dashboard: illustrates the home screen of a given research study in REDCap. Researchers may click into specific participants or metrics for further detail.

Figure 4 shows the REDCap Dashboard. When members of the research team log into REDCap to access study data, they are presented with the REDCap Dashboard. From there, they may select individual participants or individual survey results to view them in more detail.

**Figure 5:**
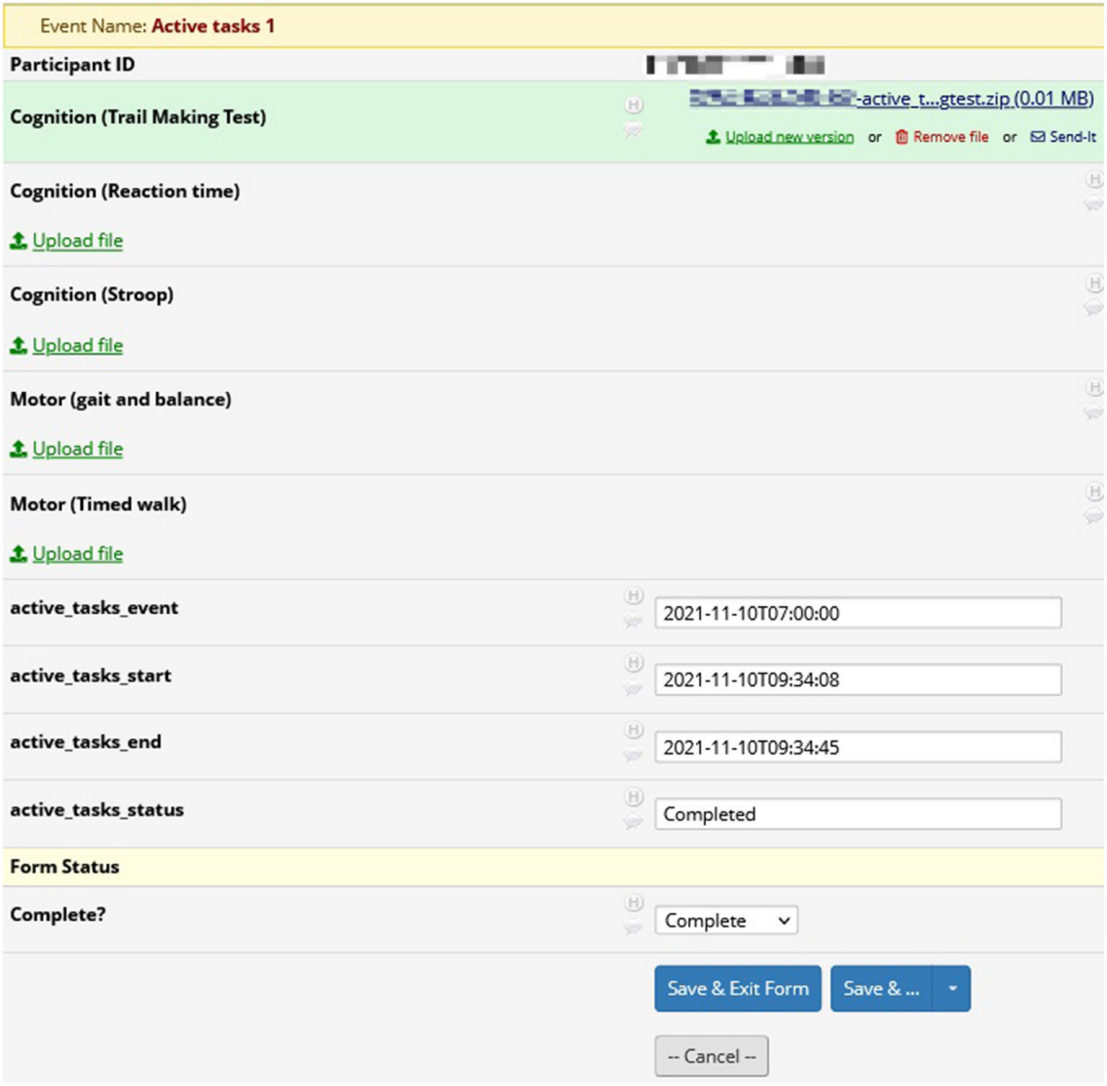
REDCap active tasks: results from active tasks in REDCap. In this example, researchers may download the results from the trail making test for analysis. Also included are the start and end times for the active task.

Figure 5 displays the page for viewing an Active Task reading from the trail making test in REDCap. Members of the research team may download and analyze the results from the Active Task. For instance, the research team will be able to see how many errors the participant made and the time taken to successfully complete the trail making Active Task. The page also includes the start and end times for the Active Task.

**Figure 6:**
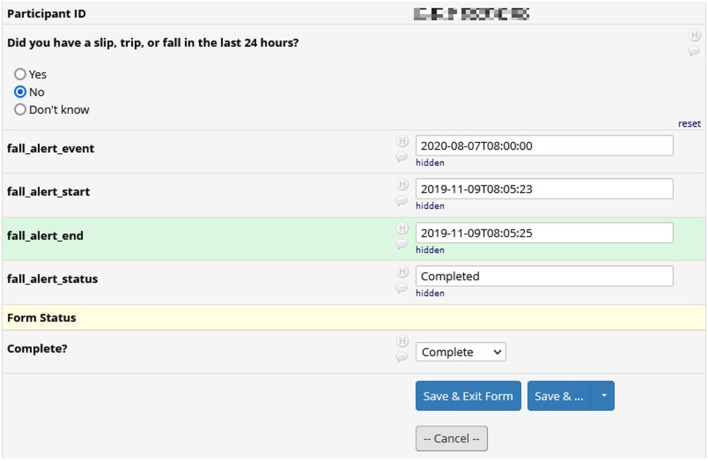
REDCap survey data: results from a survey in REDCap. Surveys were included in earlier versions of the GAPcare II protocol. Included is the participant's answer to the survey and the interval during which they completed the survey.

Figure 6 shows the results from a survey in REDCap. We included surveys during the pilot phases of the GAPcare II protocol to test the useability and acceptability of the app. Survey results in REDCap include the question asked and the participant's response, as well as information pertaining to the time of completion of the survey.

### Advantages and Challenges of This Workflow

We detail the benefits of this workflow in Table 1, as observed by the members of the research team throughout the GAPcare II study. Advantages include ease of data management and data portability, streamlined participant profile management, and ease of use once the workflow is finalized.

**Table 1:** Observed benefits of using the Apple Watch-ResearchKit-REDCap tech stack.

**Category**	**Benefits**
Consumer experience	Participants with a wide level of prior technical experience can still learn to use the app with proper training, instruction, and guidance. The Apple Watch's simple aesthetic makes it more palatable to a broader variety of participant populations
Data management	Data files are easily accessible and manageable by research staff on REDCap. Files can be downloaded for use in all major statistical languages including SAS, Stata, and R
Data management	ResearchKit allows for the collection of a broad variety of data types, including physiological data, survey data, and Active Task results
Data transfer	Data is transferred to REDCap via the API as soon as it is finalized, rather than relying on traditional, more time-consuming methods of data collection, like diaries, calendars, and paper surveys
Design and development	ResearchKit and REDCap are ready for use off the shelf for non-experts in technical development, serving to democratize access to research app development
Instrument development	Streamlined creation of forms in REDCap, coupled with the ability to edit the study app, allows for shifts in information being collected if the study needs to adapt
Portability	Participants are able to complete some study tasks from any location, instead of having to complete them in a controlled study environment
Privacy and cost	REDCap is HIPAA-compliant and free for researchers at most health systems
Remote consent	ResearchKit includes a module for electronic consent for study participants, reducing the participant burden of completing extensive paperwork
User access	Simple permissions management for members of the research team allows for new staff to be added easily to the project and for the Principal Investigator to specify limits on access, including who can download identifiable data and who can create and delete new study subjects

Some study participants had difficulty interacting with the GAPcare II study protocols during the pilot phase. We have detailed the technical challenges that we encountered during our GAPcare II study and the steps we took to overcome them in Table 2 (19).

**Table 2:** Challenges of using the Apple Watch-ResearchKit-REDCap tech stack and recommended mitigations.

**Category**	**Challenges**	**Recommendations for future use**
Data analysis	ResearchKit's data from Active Tasks and sensor data is often presented without sufficient context and, therefore, is hard to analyze and interpret without adequate understanding or experience	Create tools for converting the data to a more visualizable format, such as code to present all data on one Excel sheet. Additionally, hire a dedicated medical statistician on the team
Device access and connectivity	Participants need to have a stable internet connection in order to collect and transmit information	Screen participants for stable internet access before enrollment. Provide participants with a hotspot for the duration of the study
Digital divide based on participant demographics	Older adults with low previous technology exposure had difficulty using the app and completing Active Tasks, leading to poor data collection.	Screen older adults for technical capability before enrollment to gauge the training support needed for your project. Provide technical training as needed to eligible participants
Environmental barriers	Some Active Tasks can require mobility or access to open spaces, which are not universally availabl.	Have participants perform those Active Tasks in a research setting
Missing data	Some participants may not complete Active Tasks, surveys, or other assessments, which can lead to lapses in data collection. This can also occur if the participant is not able to charge their device	Regularly check in with study participants to ensure their continued participation in the study. Use completion metrics from REDCap to direct which participants you contact
Tech support	Less technically capable participants might need support from the research team to complete study tasks	Providing a simple, printed user manual for the app and allowing participants to contact the research team can mitigate this issue. If budgeting allows, hire a part time staff member who solely addresses study technology concerns
Technology inventory	Using multiple platforms can result in participants being identified by various IDs, (i.e., study ID, status/post username, REDCap ID) which can be overwhelming or lead to data errors	Accurate data management in REDCap forms and other software can help identify participants by differing IDs. Diligent tracking of all participant IDs from all platforms, such as on an Excel spreadsheet, can be helpful for ensuring data quality

### REDCap as a Tool to Continuously Provide Feedback to Participants

**Figure 7:**
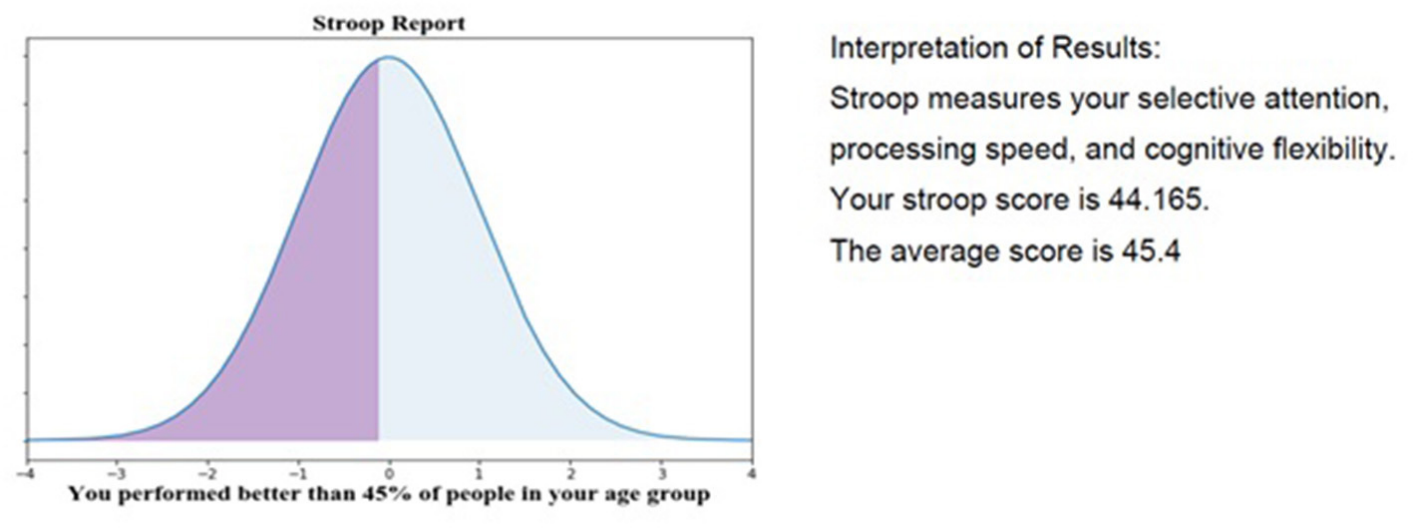
Sample analysis of data and REDCap and generated report card for study participant.

When participants completed an Active Task in RI FitTest, the resulting information was transmitted immediately to the REDCap database via the API. This data transmission enables the research team to keep participants informed about their progress in the study, potentially as a motivator for continued participation. Figure 7 displays a graph and report card generated for a participant given their performance on the Stroop Active Task. Performance updates like this can be continuously sent to participants to keep them engaged in the study and improve study retention.

### Data Management and Analyses

We performed several steps to ensure the quality and completeness of data before initiating the clinical trial. It is essential that you take these steps to ensure that you can report adherence and physical activity measures at the conclusion of your study (20).

First, we measured device adherence by generating reports using REDCap's API export feature to track daily use of the Apple Watch. Using these reports, we could distinguish if a participant had stopped transmitting data from their Apple Watch. If we noticed a lapse in data collection, we would call the participant to ask them if they were wearing the device, if the device was charged, or if they were not connected to the internet. In addition to tracking adherence with daily wear, researchers should be prepared to report the wear time per day and per week. If your study aims to understand daily activity and participants only wear the wearable in the morning when they are most sedentary, you will obtain meaningless measurements.

Additionally, before initiating recruitment the study team should perform the physical activity measures and evaluate the data obtained in REDCap to ensure that the data includes timestamps (e.g., start and stop times of each activity) and that all the necessary information is collected for analyses. For instance, by looking at the data in REDCap, you should be able to ascertain when the activity started, when it ended, if it was aborted early, whether the participant correctly followed instructions, and how well-they performed the task.

To analyze the data, the research team should be prepared to process raw unstructured data into a cleaned dataset that can be more easily analyzed. For instance, our research team wrote R code to create variables that were meaningful for our research question and then performed statistical analysis.

## Discussion

In this manuscript, we detailed an approach to building a sensor system to improve monitoring and outcome assessment in falls research. Although we used the Apple Watch, iPhone, and REDCap software for research purposes, this technology can be applied to clinical care and is an example of how consumer-facing exercise and fitness tools can be employed by clinical researchers to improve our knowledge of illness trajectories and health parameters. As detailed in Table 3, successful use of the Apple Watch as a research tool requires a multidisciplinary team of scientists experienced in collecting and analyzing large datasets, clinicians with health experience, and study personnel with the ability to provide technology training and support—particularly when conducting work in a population of older adults, who may lack prior exposure to such technologies. While older adults are willing to use wearable technologies in clinical research, Kabacińska et al. (21) they require training and support beyond what a “digital native” may need to successfully interact with the technology.

**Table 3:** Suggested study team composition for studies involving wearables.

**Expertise**	**Study tasks**
Clinical researcher/scientist	Oversee human subjects protection, provide study oversight, conduct team meetings, design study to meet research aims, communicate with sponsors, obtain funding
Research assistant	Communicate with participants, perform research tasks with participants, teach participants how to use the technology, troubleshoot technology concerns with participants
Computer scientist / biomedical engineer	Develop software, create websites, program instruments, troubleshoot technology problems, provide suggestions on improving usability of wearables
Data scientist	Data processing and quality control, create, and vet workflow for efficient data process and more accurate analyses
Data manager	Monitor for lapses in wearable use or connectivity, perform quality checks, ensure safe data storage
Developer	Program research app, make modifications depending on study findings

Researchers can leverage the same tech workflow we employed in the GAPcare II study to create a low-cost, efficient research app to collect real-time information from participants securely and efficiently. Real-world data can be a rich source of information, but researchers need to be aware of typical patterns of use, particularly high levels of initial engagement followed by a plateauing of use (22). Additionally, as wearable technologies and mobile phones and apps become more commonly used, individuals—especially older adults—may grow weary of adding to their already existing technology. Some studies using wearables have found that loss of interest and technical issues are leading causes of discontinuing study participation (23). However, the COVID-19 pandemic has made users more amenable to using mobile technology to monitor health (24).

Although we used the Apple Watch sensors to collect data relevant to falls, there are several other potential applications of this technology. The Apple Watch ECG feature has been used to detect atrial fibrillation (25), and pulse oximetry—now available through Apple Watch technology—can be used for early detection of hypoxia in chronic pulmonary diseases and in acute illnesses, such as COVID-19 (26). Active Tasks could provide novel insights into aging and the effect of health events on cognition and fitness. While we primarily used the Apple Watch for outcome assessment, prediction of fall events or cardiovascular events could also be possible as we learn more about precipitating factors in these events (27, 28).

In clinical practice and technological research, smartwatches have been used to detect heart arrhythmias—including atrial fibrillation (AFib) and sinus arrhythmia—and to prevent cryptogenic stroke (29–31). For instance, in the Apple Heart Study, the Apple Watch was used to indicate pulse irregularity indicative of AFib (32). Additionally, qualitative interviews revealed that research participants felt encouraged by hourly prompts to stand up and move, and this technology could be used to provide health nudges as behavioral interventions (33, 34). Wearable technologies can also be integrated more frequently into clinical trials research (35). The application of wearable technologies is important, as these devices are increasingly popular, with 19% of Americans currently using a wearable fitness tracker (36). Given the Apple Watch's ability to detect crucial health indicators, having an organized workflow of how to record and apply this quantitative data will be advantageous to researchers.

Several limitations should be considered when doing this work. Although we offered technical support to participants, many still felt overwhelmed by the steps required to complete tasks independently at home (19). Hands-on assistance was often needed for the population of older adults we recruited (19), considering how to provide training to participants with various skill levels and experience is essential when designing study protocols. Additionally, missing data can result from participants' underuse or incorrect use of devices, or from connection errors or programming errors at the level of the investigator. Troubleshooting these concerns is time consuming and can hinder study progress (37). Generating weekly reports of device usage using REDCap's API export feature helped us recognize when a participant's data was not getting transmitted, and we could then contact them to ensure they were wearing their watch, charging it, and correctly connecting to the internet.

Using expensive devices such as the iPhone and Apple Watch can also cause sampling bias, unless they are provided to participants free of charge. Researchers should ensure that their budget covers the upfront cost of providing this technology to their participants, and they should consider providing a return incentive. We recommend pilot testing measures with the target population before starting data collection, as vision and strength can affect ability to complete Active Tasks and could result in missing data. In some cases, researchers may then decide to choose activities that can be more easily performed by their target population or may want to reprogram activities to adapt them to the participants' abilities.

## Conclusions

Smartphones and wearable devices can be rich sources of real world data for clinical researchers interested in disease prevention, monitoring, and treatment. The workflow that we created—using ResearchKit, the Apple Watch, and REDCap—provides an example of how to design longitudinal studies using this technology. While this tech stack is straightforward to use for technologically capable members of research teams and for participants, we still recommend a multidisciplinary team with expertise in computer science, biomedical engineering, and experience performing sensor analyses. Researchers should also plan to offer hands-on support and frequent technology assistance to participants. While these digital tools are adaptable, efficient, and applicable to multiple domains of medical research, there are technical challenges that, if unaddressed, could impede researchers from harnessing the many benefits of this technology.

## Data Availability Statement

The original contributions presented in the study are included in the article/supplementary material, further inquiries can be directed to the corresponding author/s.

## Author Contributions

HD, ND, and EG designed the research project and prepared the manuscript. ND and EG oversaw the project. LJ, ML, DS, KF, and TM made contributions to the preparation and revision of the manuscript. All authors contributed to manuscript revision, read, and approved the submitted version.

## Funding

This project receives funding from the National Institute on Aging via a K76 grant, grant K76AG059983. Apple Watches were supplied for study purposes from Apple through Apple's Investigator Support Program.

## Conflict of Interest

The authors declare that the research was conducted in the absence of any commercial or financial relationships that could be construed as a potential conflict of interest.

## Publisher's Note

All claims expressed in this article are solely those of the authors and do not necessarily represent those of their affiliated organizations, or those of the publisher, the editors and the reviewers. Any product that may be evaluated in this article, or claim that may be made by its manufacturer, is not guaranteed or endorsed by the publisher.
